# HIV Testing and Risk Behaviors Among Gay, Bisexual, and Other Men Who Have Sex with Men — United States

**Published:** 2013-11-29

**Authors:** Gabriela Paz-Bailey, H. Irene Hall, Richard J. Wolitski, Joseph Prejean, Michelle M. Van Handel, Binh Le, Michael LaFlam, Linda J. Koenig, Maria Corazon Bueno Mendoza, Charles Rose, Linda A. Valleroy

**Affiliations:** Div of HIV/AIDS Prevention, National Center for HIV/AIDS, Viral Hepatitis, STD, and TB Prevention, CDC

The burden of human immunodeficiency virus (HIV) is high among gay, bisexual, and other men who have sex with men (MSM) (1). High HIV prevalence, lack of awareness of HIV-positive status, unprotected anal sex, and increased viral load among HIV-positive MSM not on antiretroviral treatment contribute substantially to new infections among this population. CDC analyzed data from the National HIV Surveillance System (NHSS) to estimate the percentage of HIV diagnoses among MSM by area of residence and data from the National HIV Behavioral Surveillance System (NHBS) to estimate unprotected anal sex in the past 12 months among MSM in 2005, 2008, and 2011; unprotected discordant anal sex at last sex (i.e., with a partner of opposite or unknown HIV status) in 2008 and 2011; and HIV testing history and the percentage HIV-positive but unaware of their HIV status by the time since their last HIV test in 2011. This report describes the results of these analyses. In all but two states, the majority of new HIV diagnoses were among MSM in 2011. Unprotected anal sex at least once in the past 12 months increased from 48% in 2005 to 57% in 2011 (p<0.001). The percentage engaging in unprotected discordant anal sex was 13% in 2008 and 2011. In 2011, 33% of HIV-positive but unaware MSM reported unprotected discordant anal sex. Among MSM with negative or unknown HIV status, 67% had an HIV test in the past 12 months. Among those tested recently, the percentage HIV-positive but unaware of their infection was 4%, 5%, and 7% among those tested in the past ≤3, 4–6, and 7–12 months, respectively. Expanded efforts are needed to reduce HIV risk behaviors and to promote at least annual HIV testing among MSM.

What is already known on this topic?

Although men who have sex with men (MSM) are a small proportion of the population, MSM represent the majority of persons diagnosed with human immunodeficiency virus (HIV) in the United States.

What is added by this report?

Unprotected anal sex increased among MSM from 2005 to 2011; unprotected discordant anal sex was the same in 2008 and 2011. In 2011, one third of HIV-positive MSM who did not know they were infected with HIV reported recent unprotected anal sex with a partner of HIV-negative or unknown status, compared with 13% of HIV-positive aware and 12% of HIV-negative MSM. Only 67% of sexually active MSM reported getting an HIV test in the past year.

What are the implications for public health?

Expanded efforts are needed to reduce HIV risk behaviors and to promote at least annual HIV testing among MSM. Health-care providers and public health officials should work to ensure that 1) sexually active, HIV-negative MSM are tested for HIV at least annually (providers may recommend more frequent testing, for example every 3–6 months); 2) HIV-negative MSM who engage in unprotected sex receive risk-reduction interventions; and 3) HIV-positive MSM receive HIV care, treatment, and prevention services.

Data reported through June 2012 to NHSS were used to estimate* HIV diagnoses among MSM by area of residence in 2011. Data from NHBS† were used to describe adjusted trends in unprotected anal sex§ in the past 12 months among MSM in 2005, 2008, and 2011.¶ Data from 2008 and 2011 were used to calculate the prevalence of unprotected discordant anal sex** at last sex. Chi-square tests†† were used to evaluate differences between 2008 and 2011 by HIV status, race/ethnicity, and age. Data from 2011 were used to evaluate the difference in the percentage engaging in unprotected discordant anal sex at last sex among HIV-positive aware,§§ HIV-positive unaware, and HIV-negative MSM. Adjusted¶¶ prevalence ratios (APRs) and 95% confidence intervals (CIs) are presented. Data from 2011 were used to assess HIV testing history after excluding self-reported HIV-positive MSM, and the percentage HIV-positive but unaware, by time since the last HIV test.

In 2011, MSM accounted for at least half of persons diagnosed with HIV in all but two states (Figure 1). The percentage of MSM reporting unprotected anal sex at least once in the past 12 months increased from 2005 to 2011, from 48% in 2005, to 54% in 2008, and 57% in 2011 (p<0.001). The trend was statistically significant among self-reported HIV-negative or unknown status MSM (47%, 54%, and 57%, respectively; p<0.001), but not statistically significant for self-reported HIV-positive MSM (55%, 57%, and 62%, respectively; p=0.054) (Table 1).

The percentage of MSM engaging in unprotected discordant anal sex at last sex was 13% in both 2008 and 2011 (Table 2). In 2011, 33% of HIV-positive but unaware MSM had unprotected discordant anal sex at last sex. This percentage was more than twice as high as the percentage among those who were HIV-positive aware (13%) (APR = 2.2; CI = 1.7–2.9; p<0.001) or HIV-negative (12%) (APR = 2.8; CI = 2.2–3.5; p<0.001).

Among HIV-negative or unknown status MSM, 67% reported testing for HIV in the past 12 months. A higher percentage tested in the past 3 months (31%) than in the past 4–6 months (17%) or in the past 7–12 months (19%) (Figure 2). The percentage HIV-positive but unaware was 5% among those who tested in the past 12 months: 4%, 5%, and 7% among those tested ≤3, 4–6, and 7–12 months ago, respectively (Figure 3).

## Editorial Note

Although MSM are a small proportion of the population, they represent the majority of persons diagnosed with HIV in nearly every U.S. state. Unprotected anal sex in the last 12 months increased nearly 20% among MSM from 2005 to 2011. MSM unaware of their HIV-positive status were more than twice as likely to have unprotected discordant anal sex at last sex as MSM who were either HIV-negative or HIV-positive aware. Only 67% of MSM had tested for HIV in the past 12 months.

Unprotected anal sex is a high-risk practice for HIV infection, with receptive anal sex having the highest risk (2). Unprotected anal sex also places MSM at risk for other sexually transmitted infections such as syphilis, chlamydia, and gonorrhea. Although condoms can reduce the risk for HIV transmission, they do not eliminate risk and often are not used consistently (3). Some MSM attempt to decrease their HIV risk by engaging in unprotected sex only with partners perceived to have the same HIV status as their own. However, this practice is risky, especially for HIV-negative MSM, because MSM with HIV might not know or disclose that they are infected and men’s assumptions about the HIV status of their partners can be wrong (2).

The reasons for the increase in unprotected anal sex are not fully known but might partially reflect the adoption of presumed risk-reduction strategies, such as engaging in unprotected sex only with partners perceived to have the same HIV status as one’s own (4). The fact that the same percentage of MSM engaged in unprotected discordant anal sex at last sex in 2008 and 2011 supports this hypothesis.

Among MSM participating in the National HIV Behavioral Surveillance System (NHBS) in 2011, 18% were HIV-positive (5). Awareness of HIV-positive status among HIV-infected MSM increased from 56% in 2008 to 66% in 2011 in the 20 cities participating in NHBS (5). However, one third of HIV-positive MSM in NHBS did not know that they were infected with HIV (5), and a high percentage of them reported recent unprotected discordant anal sex with a partner of HIV-negative or unknown status. CDC found that MSM who were HIV-positive but unaware were more than two times more likely to engage in unprotected discordant anal sex, compared with HIV-positive aware or HIV-negative MSM. Persons aware of their infection are less likely to transmit the virus (6), and HIV testing is an essential first step in the care and treatment of those who are HIV-positive. HIV treatment can lower viral load, improving health outcomes and reducing the likelihood of HIV transmission. About eight transmissions would be averted for every 100 persons newly aware of their infection as a result of HIV treatment and reductions in risk behavior (6). CDC recommends that persons at high-risk for HIV, such as sexually active MSM, be tested at least annually (7,8). However, in this analysis one third of MSM had not tested for HIV in the past 12 months. Increased use of HIV testing and more frequent testing among sexually active MSM might reduce the number of men unaware of their HIV status and reduce HIV transmission.

The findings in this report are subject to at least two limitations. First, NHBS data are from MSM who were recruited at venues in large cities. Thus, results might not be generalizable to all MSM. Second, except for HIV testing results, analyses were based on self-reported data and might be subject to social desirability and recall bias.

Sexually active MSM should be tested at least annually for HIV and other sexually transmitted infections. Sexually active MSM can take steps to make sex safer such as choosing less risky behaviors, using condoms consistently and correctly if they have vaginal or anal sex, reducing the number of sex partners, and if HIV-positive, letting potential sex partners know their status (2). For some MSM at high risk, taking preexposure or postexposure prophylaxis can reduce risk (9). Health-care providers and public health officials should work to ensure that 1) sexually active, HIV-negative men are tested for HIV at least annually (providers may recommend more frequent testing, for example every 3–6 months); 2) HIV-negative MSM who engage in unprotected sex receive risk-reduction interventions; and 3) HIV-positive MSM receive HIV care, treatment, and prevention services. Reducing the burden of HIV among MSM is fundamental to reducing HIV infection in this country.

## Figures and Tables

**FIGURE 1:**
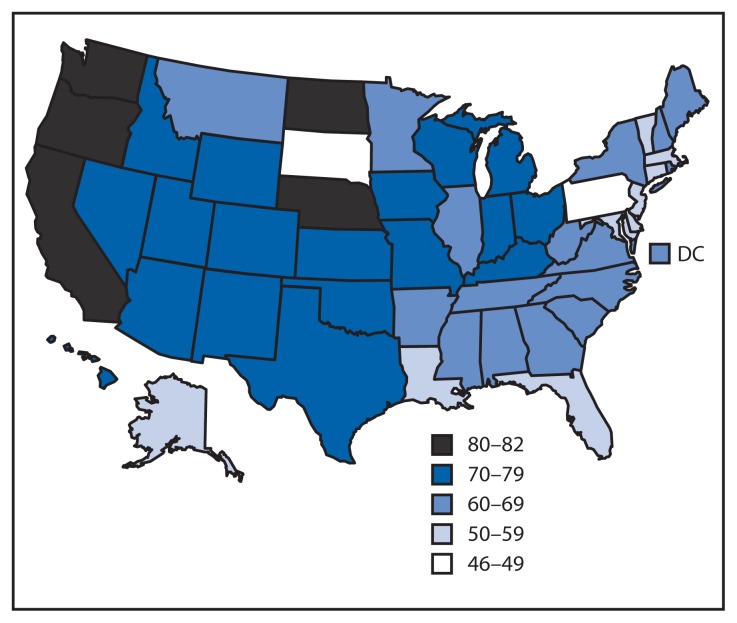
Estimated percentage of persons diagnosed with HIV with infection attributed to male-to-male contact or male-to-male contact and injection drug use, by area of residence — National HIV Surveillance System, United States, 2011

**FIGURE 2:**
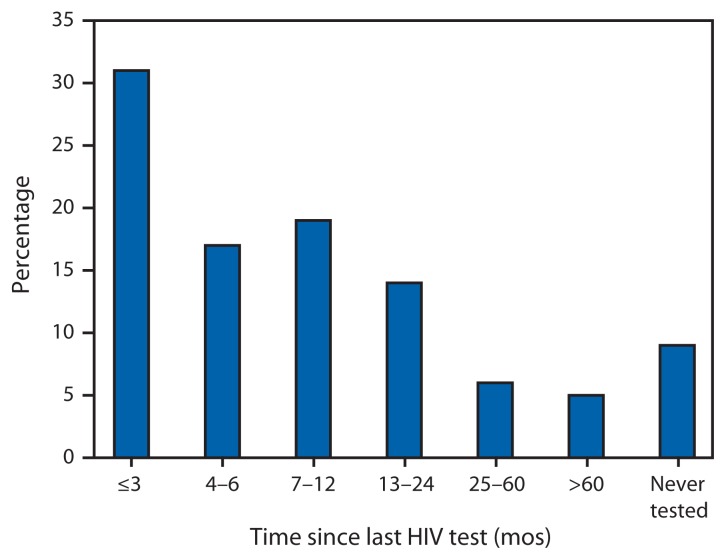
Time since last human immunodeficiency virus (HIV) test among men who have sex with men who reported negative or unknown HIV status,* — National HIV Behavioral Surveillance System, United States, 2011^†^ * Includes respondents who reported their last HIV test result was negative, indeterminate, did not receive test results, did not know the results, or had never been tested. ^†^ N = 7,312; excludes 76 respondents missing data for time of HIV test.

**FIGURE 3:**
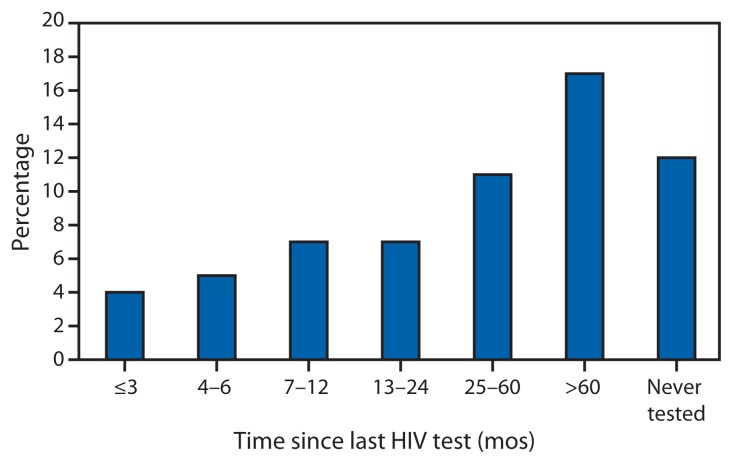
Percentage who were human immunodeficiency virus (HIV)-positive unaware among men who have sex with men who reported negative or unknown HIV status, by time since last HIV test — National HIV Behavioral Surveillance System, United States, 2011* * N = 7,312; excludes 76 respondents missing data for time of HIV test. Bars represents percentage testing positive in the survey among men who have sex with men who reported having had an HIV test at each time interval.

**TABLE 1:** Number and percentage of men who have sex with men who reported unprotected* anal sex with a male partner in the past 12 months, by self-reported human immunodeficiency virus (HIV) status — National HIV Behavioral Surveillance System, United States, 2005, 2008, and 2011†

	2005			2008			2011			
				
Characteristic	No. in sample	No.	(%)	No. in sample	No.	(%)	No. in sample	No.	(%)	p-value§
**Self-reported HIV-positive**										
**Overall**	**1,441**	**796**	**(55)**	**1,101**	**623**	**(57)**	**1,244**	**769**	**(62)**	**0.054**
**Race/Ethnicity**										
Black, non-Hispanic	296	140	(47)	269	137	(51)	417	235	(56)	0.026
Hispanic¶	285	146	(51)	228	124	(54)	262	156	(60)	0.198
White, non-Hispanic	744	446	(60)	526	320	(61)	488	332	(68)	0.051
Other/Multiple races**	103	59	(57)	78	42	(54)	72	43	(60)	0.771
**Age group (yrs)**										
18–24	49	26	(53)	79	41	(52)	143	78	(55)	0.776
25–29	98	64	(65)	123	77	(63)	167	116	(69)	0.246
30–39	569	342	(60)	326	207	(63)	316	227	(72)	0.002
≥40	725	364	(50)	573	298	(52)	618	348	(56)	0.092
**Self-reported HIV-negative or unknown status** ††										
**Overall**	**10,016**	**4,693**	**(47)**	**8,152**	**4,394**	**(54)**	**8,009**	**4,546**	**(57)**	**<0.001**
**Race/Ethnicity**										
Black, non-Hispanic	1,732	697	(40)	1,919	952	(50)	2,068	1,003	(49)	0.113
Hispanic¶	2,677	1,265	(47)	2,004	1,138	(57)	2,145	1,340	(62)	<0.001
White, non-Hispanic	4,506	2,235	(50)	3,498	1,921	(55)	3,177	1,840	(58)	<0.001
Other/Multiple races**	993	443	(45)	725	380	(52)	600	350	(58)	<0.001
**Age group (yrs)**										
18–24	2,186	996	(46)	1,992	1,133	(57)	2,209	1,302	(59)	<0.001
25–29	1,813	912	(50)	1,588	944	(59)	1,583	965	(61)	<0.001
30–39	3,310	1,646	(50)	2,236	1,232	(55)	1,874	1,119	(60)	0.003
≥40	2,707	1,139	(42)	2,336	1,085	(46)	2,343	1,160	(50)	<0.001
**Total**	**11,457**	**5,489**	**(48)**	**9,253**	**5,017**	**(54)**	**9,253**	**5,315**	**(57)**	**<0.001**

* Neither the respondent nor his sex partner used a condom all the time.

† Percentages might not add to 100 because of rounding; numbers might not add to total because of missing data.

§ Adjusted p-values for the 2005 to 2011 trend; all models include year, age, race/ethnicity, and city and interactions for year × age and year × race/ethnicity. Interactions for year × age and year × race/ethnicity were not statistically significant, suggesting that no overall difference in trend existed between race/ethnicity categories, likewise for age categories. P<0.05 is considered statistically significant.

¶ Respondents of Hispanic ethnicity might be of any race.

** Other races include American Indian/Alaska Native, Asian, Native Hawaiian/other Pacific Islander, and mixed race.

†† Includes respondents who reported their last HIV test result was negative, indeterminate, did not receive test results, did not know the results, or had never been tested.

**TABLE 2:** Number and percentage of men who have sex with men who reported unprotected* anal sex at last sex with a male partner of human immunodeficiency virus (HIV) discordant or unknown status, by HIV status of the participant — National HIV Behavioral Surveillance System, United States, 2008 and 2011†

	2008			2011			
			
Characteristic	No. in sample	No.	(%)	No. in sample	No.	(%)	p-value§
**Self-reported HIV-positive**							
***HIV-positive aware***¶ ***with a partner of HIV-negative or unknown status***							
**Overall**	**882**	**139**	**(16)**	**1,032**	**139**	**(13)**	**0.16**
**Race/Ethnicity**							
Black, non-Hispanic	219	36	(16)	357	47	(13)	0.28
Hispanic**	190	29	(15)	216	41	(19)	0.32
White, non-Hispanic	410	69	(17)	394	42	(11)	0.01
Other/Multiple races††	63	5	(8)	60	9	(15)	0.22
**Age group (yrs)**							
18–24	62	8	(13)	123	15	(12)	0.89
25–29	95	15	(16)	139	26	(19)	0.56
30–39	256	50	(20)	254	39	(15)	0.21
>40	469	66	(14)	516	59	(11)	0.21
**Self-reported HIV-negative or unknown status**							
***HIV-positive unaware***§§ ***with a partner of HIV-negative or unknown status***							
**Overall**	**676**	**201**	**(30)**	**521**	**174**	**(33)**	**0.18**
**Race/Ethnicity**							
Black, non-Hispanic	314	82	(26)	307	97	(32)	0.13
Hispanic**	163	44	(27)	124	44	(35)	0.12
White, non-Hispanic	138	52	(38)	65	24	(37)	0.92
Other/Multiple races††	61	23	(38)	24	8	(33)	0.71
**Age group (yrs)**							
18–24	135	33	(24)	129	41	(32)	0.18
25–29	128	40	(31)	104	29	(28)	0.58
30–39	212	65	(31)	127	51	(40)	0.07
≥40	201	63	(31)	161	53	(33)	0.75
***HIV-negative with partner of HIV-positive or unknown status***							
**Overall**	**6,591**	**734**	**(11)**	**6,867**	**806**	**(12)**	**0.27**
**Race/Ethnicity**							
Black, non-Hispanic	1,346	164	(12)	1,551	198	(13)	0.64
Hispanic**	1,676	249	(15)	1,885	260	(14)	0.37
White, non-Hispanic	2,959	271	(9)	2,879	291	(10)	0.22
Other/Multiple races††	605	49	(8)	538	53	(10)	0.30
**Age group (yrs)**							
18–24	1,691	196	(12)	1,930	236	(12)	0.56
25–29	1,306	143	(11)	1,382	141	(10)	0.53
30–39	1,761	187	(11)	1,597	191	(12)	0.22
≥40	1,833	208	(11)	1,958	238	(12)	0.44
**Total**	**8,149**	**1,074**	**(13)**	**8,420**	**1,119**	**(13)**	**0.83**

* Neither the respondent nor his sex partner used a condom all the time.

† Percentages might not add to 100 because of rounding; numbers might not add to total because of missing data.

§ Chi-square p-value for comparison of 2008 and 2011 percentages. P<0.05 is considered statistically significant.

¶ Respondents with a confirmed positive HIV test result in the survey who reported having previously tested positive for HIV.

** Respondents of Hispanic ethnicity might be of any race.

†† Other races include American Indian/Alaska Native, Asian, Native Hawaiian/other Pacific Islander, and mixed race.

§§ Includes respondents with a confirmed positive HIV test result in the survey who reported their last HIV test result was negative, indeterminate, did not receive test results, did not know the results, or had never been tested.

## Footnotes

* Estimated numbers of HIV diagnoses resulted from statistical adjustment that accounted for reporting delays and missing transmission category but not for incomplete reporting. Diagnoses data are used to describe the geographic distribution of the HIV burden among MSM.

† NHBS monitors HIV-associated behaviors and HIV prevalence within selected metropolitan statistical areas (MSAs) with high acquired immunodeficiency syndrome (AIDS) prevalence among three populations at high risk for HIV infection: MSM, injection drug users, and heterosexual adults at increased risk for HIV infection. Data for NHBS are collected in annual rotating cycles. All NHBS participants must be aged ≥18 years, live in a participating MSA, and be able to complete a behavioral survey in English or Spanish. MSM participants were recruited using venue-based sampling. The first MSM cycle of NHBS in 2003–2005 (referred to as 2005 in this report) included the following cities: Atlanta, Georgia; Baltimore, Maryland; Boston, Massachusetts; Chicago, Illinois; Denver, Colorado; Fort Lauderdale, Florida; Houston, Texas; Los Angeles, California; Miami, Florida; Newark, New Jersey; New York City, New York; Philadelphia, Pennsylvania; San Diego, California; San Francisco, California; and San Juan, Puerto Rico. The second MSM cycle of NHBS in 2008 included all the cities in the first cycle except Fort Lauderdale, Florida; plus the following cities: Washington, DC; Dallas, Texas; Detroit, Michigan; New Orleans, Louisiana; Nassau-Suffolk, New York; St. Louis, Missouri; and Seattle, Washington. The third MSM cycle of NHBS included all the cities in the second cycle except for St. Louis.

§ Unprotected anal sex was defined as sex without a condom with a male partner at least once in the 12 months before the survey interview. The outcome is reported for self-reported HIV-positive and self-reported HIV-negative or unknown-status MSM. Self-reported negative and unknown-status MSM are grouped together to represent the group “at risk” for HIV infection based on self-reported status. Persons of unknown status include: last HIV test results were indeterminate, did not receive test results, did not know the results, or had never been tested. The analysis included all MSM participating in NHBS irrespective of whether they had an HIV test through NHBS. Men who consented to and completed the survey and reported having a male sex partner in the past year were included in the analyses. All cities that participated in any of the three cycles of NHBS among MSM were included. Because the studies used different geographic eligibility criteria with slightly different cities participating in each cycle, a sensitivity analysis limited to the 14 cities that participated in all three cycles was conducted and found similar results.

¶ Generalized estimating equations using a robust variance estimate and assuming a Poisson model were used to test if a linear trend exists between 2005, 2008, and 2011 in the percentage of MSM that had unprotected anal sex at least once with a male partner in the past 12 months. All models included year, age, race/ethnicity, and city and interactions for year × age and year × race. Year was treated as a continuous variable. P-values for the 2005 to 2011 trend were calculated (Table 1); p<0.05 was considered statistically significant.

** Data from 2008 and 2011 for MSM with a valid HIV test result (positive or negative) were used to describe unprotected discordant anal sex, defined as not using a condom at last sex with a male partner of opposite or unknown HIV status. Based on HIV-test results, the analysis subgroups for the first outcome (self-reported positive and self-reported negative or unknown status MSM) are further divided into HIV-positive aware, HIV-positive unaware, and HIV-negative MSM. HIV-positive aware MSM are defined as self-reported HIV-positive MSM with a confirmed positive HIV test result in the NHBS survey. HIV-positive unaware MSM are defined as MSM with a confirmed positive HIV test result in the NHBS survey who reported their last HIV test result was negative, indeterminate, did not receive test results, did not know the results, or had never been tested. HIV-negative MSM are defined as self-reported negative or unknown HIV status participants with an HIV-negative test result in the NHBS survey. Data from 2005 were excluded from this analysis since HIV testing was only conducted in five cities.

†† Because no statistically significant difference (p<0.05) was found in the percentage engaging in unprotected discordant sex between years, a multivariate analysis was not conducted for this outcome (Table 2).

§§ Respondents with a confirmed positive HIV test result in NHBS who reported having previously tested positive for HIV were considered to be aware of their infection. Those with a confirmed positive HIV test result in NHBS, who reported previously testing negative, not knowing their last test result or never testing, were considered unaware of their HIV status.

¶¶ Generalized estimating equations using a robust variance estimate and assuming a Poisson model were used to determine the associations between unprotected discordant sex and HIV status (using HIV-positive unaware as the reference category) after adjusting for race/ethnicity, age, and city.
